# Lean Six Sigma Redesign of a Process for Healthcare Mandatory Education in Basic Life Support—A Pilot Study

**DOI:** 10.3390/ijerph182111653

**Published:** 2021-11-06

**Authors:** Anne Dempsey, Ciara Robinson, Niamh Moffatt, Therese Hennessy, Annmarie Bradshaw, Sean Paul Teeling, Marie Ward, Martin McNamara

**Affiliations:** 1 Beacon Hospital, Sandyford, D18 AK68 Dublin, Ireland; ciara.robinson@beaconhospital.ie (C.R.); niamh.moffatt@beaconhospital.ie (N.M.); therese.hennessy@beaconhospital.ie (T.H.); annmarie.bradshaw@beaconhospital.ie (A.B.); 2 UCD Centre for Interdisciplinary Research, Education & Innovation in Health Systems, School of Nursing, Midwifery & Health Systems, University College Dublin, D04 V1W8 Dublin, Ireland; sean.p.teeling@ucd.ie (S.P.T.); martin.mcnamara@ucd.ie (M.M.); 3 Centre for Person-Centred Practice Research Division of Nursing, School of Health Sciences, Queen Margaret University Drive, Queen Margaret University, Musselburgh EH21 6UU, UK; 4 Centre for Innovative Human Systems, School of Psychology, Trinity College, The University of Dublin, D02 PN40 Dublin, Ireland; marie.ward@tcd.ie

**Keywords:** Basic Life Support, training, education, lean six sigma, mandatory education, process improvement, cardiac pulmonary resuscitation, education, virtual learning environment, blended learning in healthcare, American Heart Association

## Abstract

Healthcare staff are required to undertake mandatory training programs to ensure they maintain key clinical competencies. This study was conducted in a private hospital in Ireland, where the processes for accessing mandatory training were found to be highly complex and non-user friendly, resulting in missed training opportunities, specific training license expiration, and underutilized training slots which resulted in lost time for both the trainers and trainees. A pilot study was undertaken to review the process for accessing mandatory training with a focus on the mandatory training program of Basic Life Support (BLS). This was chosen due to its importance in patient resuscitation and its requirement in the hospital achieving Joint Commission International (JCI) accreditation. A pre- and post-team-based intervention design was used with Lean Six Sigma (LSS) methodology employed to redesign the process of booking, scheduling, and delivery of BLS training leading to staff individual BLS certification for a period of two years. The redesign of the BLS training program resulted in a new blended delivery method, and the initiation of a pilot project led to a 50% increase in the volume of BLS classes and a time saving of 154 h 30 min for staff and 48 h 14 min for BLS instructors. The success of the BLS process access pilot has functioned as a platform for the redesign of other mandatory education programs and will be of interest to hospitals with mandatory training requirements that are already facing healthcare challenges and demands on staff time.

## 1. Introduction

This study was undertaken in a large private hospital in Dublin, Ireland between 2020 and 2021 during a global COVID-19 pandemic. The hospital team comprises over 1800 multidisciplinary healthcare staff across all grades and professions. The hospital supports an onsite education and training academy with links to a university-accredited Lean Six Sigma (LSS) education and training program [1] to facilitate staff education and training in the use of LSS, which the hospital uses as an improvement methodology. LSS use has demonstrated its usefulness in improving process efficiency and has been shown to have a positive impact on patient outcomes, and patient and staff experiences of care [2,3].

Lean describes the philosophy of the Toyota Production System (TPS) [4,5,6,7] which was developed in the car manufacturing industry. Syrett and Lammiman [8] claim that Lean can be seen as a ‘coherent philosophy’ that introduces new ways of working or doing things that can be considered ‘leanness’. Lean is based largely on Taiichi Ohno’s [9] insights, where production activities are either classified as value-adding or waste (non-value-adding), with the purpose to increase the proportion of value-adding activities in a process using methods such as pull, flow, standardized work, leveling, and continuous improvements. Value is based on the end customers’ perception giving an outside reference to a process [4].

Six Sigma is a data-driven improvement methodology designed to improve process capability and enhance process throughput through the creation of improvement projects [10,11,12]. It has a strong focus on data and facilitates problem-solving using the Define, Measure, Analyze, Improve, Control (DMAIC) framework [13,14]. Within DMAIC stakeholder or ‘customer’ engagement is sought from the outset at the Define stage. This stage aims to create value for the customer by identifying issues that need solutions early on [15], utilizing the extensive knowledge base of customers and other stakeholders [16]. Both Lean and Six Sigma have a strong focus on the customer, the employee, management support, and teamwork [17].

A hybrid of Lean and Six Sigma as LSS appears in the healthcare literature from 2010 onwards [18] following Lean and Six Sigma integration for project delivery from early 2002 and increased use by 2008. One of the key strengths of LSS is that it seeks to find the ‘root cause’ of problems in a process, through real-time observational data collection [19,20,21,22]. The integrated approach of LSS has demonstrated improvement in process efficiency, optimizing resources, and increasing customer satisfaction while improving profits and curtailing cost [9]. LSS use in healthcare has evidenced improvements in areas such as length of stay, reduced hospital waiting times [23,24,25], improved access to diagnostics and treatment [26,27,28], and reduction in healthcare costs and waste [29].

Mandatory training is provided in every healthcare organization internationally for staff, the type of training varies depending on whether the healthcare setting is private or public. Mandatory training topics include, but are not limited to, Fire Safety, Child Protection, and Manual handling. Every healthcare organization has mandated training that they are legally required to provide depending on the national health and safety regulations, healthcare standards, and accreditation processes in place. For example, the study site is a private hospital that is subject to health and safety authority regulations, the health and safety at work act, and JCI which provides private hospital accreditation [30]. Internationally any private hospital accredited with JCI will have mandatory education examined through the Staff, Qualification, and Education standard (SQE). This ensures that all training is in date with a specific focus on Basic Life Support (BLS). Staff availability to attend can be a barrier to mandatory training attendance due to increasing demands on staff time within the healthcare system. However, an efficient system and process for mandatory training is an enabler for staff to complete their training within the required timeframe. In the study site, the mandatory training programs for staff ranked in order of importance at an organizational level were identified as manual handling training, which is instruction and training on how to safely handle and move objects and people [31]; and BLS training. Traditionally, training for both was provided in the classroom setting. However, over the last two years, this training has been provided asynchronously online nationally in Ireland due to the global COVID-19 pandemic. This allows mandatory training to be completed at staff members’ convenience via external or internally developed virtual learning environments (VLE).

## 2. Background

The study site has been accredited by JCI for the last fifteen years. The first BLS training was conducted in 2006 on the opening of the hospital. Quality Improvement Projects (QIPs) have been continuous since the hospital commenced service delivery. In 2017 the hospital adopted the LSS methodology for QIPs within the organization which is supported through the onsite education and training academy [2]. This study highlights how challenges in delivering mandatory training had arisen over the past two years due to the rapid growth of the organization, a 28% increase in the number of clinical staff, and the type of education and training classes to be held for mandatory training. COVID-19 restrictions, regulations, and the capacity to release staff time for mandatory training were also drivers for this project. Our team chose to focus this study on BLS training for several reasons. Firstly, BLS training saves lives [32]. It is important that all registered nurses and designated health and social care professionals (HSCPs) maintain their BLS certification every two years for safe patient care and to keep up to date with their mandatory training requirements. The method of delivery at the initiation of this project was a standardized national approach supported by the American Heart Association (AHA) and the Irish Heart Foundation (IHF). The BLS training was a face-to-face attendance in a classroom environment, with video-assisted learning, continuous skills assessment, and a formative Multiple-Choice Questions (MCQs) assessment at the end of the class to consolidate learning. BLS training equips staff with the requisite skills and knowledge to provide early resuscitation techniques which enhance a person’s chances of survival [33]. As BLS was the most time-consuming process of all mandatory training, and any time released could be reinvested into direct patient–clinician contact time, it was therefore seen as an area for improvement. Finally, BLS was identified as the key focus for mandatory training due to the requirement to meet JCI accreditation.

In line with our organizational approach to improvement, we used the LSS methodology to redesign the current process for BLS training. There was a lack of published research specific to the use of both LSS methodologies concerning mandatory training to draw on for this study. However, the literature reviewed by our team highlighted to us how the LSS methodology and concepts can significantly contribute to identifying non-value adding (NVA) and enhance process improvement [2,3,34]. Additionally, it highlighted that cross-functional team training in healthcare supports positive improvement intervention results [35,36]. These findings assisted the team in reviewing the study site’s BLS training processes as the training is team-based and solidifies the curriculum design of the training. A LSS-driven project constitutes the scoping, planning, and implementation events required to improve a process, however big that process is [35,36].

## 3. Methods

The objective of this study was to redesign and improve the process of BLS scheduling, training, and certification within the study site. We used LSS methodology as it has demonstrated success in healthcare settings in both clinical and non-clinical areas and had been used successfully within the study site [29] previously. We asked the question ‘Can the application of LSS in healthcare mandatory training improve access to and improve the volume of classes, and release nursing time for care? To answer this question, we used a pre- and post-intervention study design [34,35], that measures the occurrence of an outcome before and after a particular intervention is implemented. Pre- and post-intervention studies involve the measurement of the variables of interest before and after the intervention in the same study site, on the assumption that any difference in measurement between ‘before’ and ‘after’ to the intervention [37]; in this case, the intervention of a LSS improvement project. Although this design has the limitation of ascribing with certainty results to an intervention [37], the use of this design has been widely used to evaluate LSS in healthcare [17,29,38,39,40,41]. It was inherent in our study design that any outcomes from redesigning the BLS training process could then be extrapolated and applied to all other mandatory training within the organization where relevant. BLS requires renewal every two years and is mandatory for JCI for all registered nurses and designated HSCPs to maintain their BLS skills.

The project was co-led by an interdisciplinary team of five staff. The team represents a broad spectrum of disciplines across the organization to include a Emergency Department (ED) Clinical Nurse Manager, a Patient Services Team Lead, a Clinical Practice Support nurse (Education department)/Clinical Analyst from Patient Safety and Quality Innovation departments, a Patient Safety and Quality Innovation Analyst, and a Corporate Administrator. This approach mirrors the LSS approach of cross-functional teams and it is seen as an enabler of successful process improvement outcomes [17,41]. Within the study site, at the start of this project, the time from the scheduling of to actual completion of training and certification in BLS took 5 h as opposed to 2 h from the scheduling to certification in Manual handling.

Aij and Tunissen [42] discuss the role of Lean leaders in process improvements and their key attributes which include clear and supportive communication, engaging, and creating an improvement culture. As a team working collectively we were aware of the importance of these when undertaking this improvement work. We worked collectively and utilized person-centered approaches to our own teamwork and our engagement with our stakeholders. Person-centered approaches speak to every person involved in both the delivery and receipt of at all stages of the process [42]. This has been shown to be synergistic with LSS approaches to improvement [43] and to be effective when combined in making healthcare process improvements [43,44]. Table 1 indicates specific LSS improvement tools that were employed within the LSS approach. We now discuss each stage of our methods under the corresponding DMAIC framework section.

### 3.1. Stage 1: Define

For our project, we focused on the recertification of BLS for directly employed clinical staff only as they have direct patient contact daily. This cohort is most likely to apply BLS skills to save a life, therefore, enhancing patient safety and ensuring good clinical outcomes for patients. The scheduling and booking process for BLS along with the method of delivery was also in our project scope.

The define phase began with a team meeting to discuss our project objective to standardize and optimize the delivery of mandatory training. As outlined, we had decided to focus our improvement on BLS, so we began by drafting a team project charter, which served as an outline for the intended improvement [36] that was agreed by all participating team members. Anecdotally we were aware that staff were not attending BLS training before their license expiry date. Baseline data evidenced that in 2018, 62% of clinical staff who completed BLS training did so after their expiry date, in 2019 it was 39% and in 2020 it was 54%. There was also difficulty in meeting increased demand for BLS training, concurrent with the aforementioned increase in staff numbers. This was due to the prioritization of clinical work time and within the process of BLS itself, time constraints in all areas of BLS completion, including scheduling, booking, and attending BLS training. As the demand for BLS training increased (Table 2) it continued to generate a large amount of administration workload for the Education Team/Clinical Nurse Managers and this was further complicated by COVID-19 restrictions which reduced BLS class capacity by 33%. The time to attend each BLS class was at this point in time 4 h 30 min.

To enable a better understanding of the complex process, we began with a SIPOC tool used to give a high-level view of a process rather than a micro level view as a precursor to further study [35] which also allowed us to examine the process to become certified in BLS from a staff member’s perspective. Using the SIPOC process map tool enabled the team to get a high-level overview of the process [15]. It enabled us to better answer key questions about how to sequence our project by identifying the process steps and key stakeholders who suppliers or customers of the process were.

Identification of key stakeholders was completed by using a stakeholder analysis tool [45,46]. The Key Stakeholders identified from this were the: Chief Operations Officer, Director of Human Resources, Director of Nursing, and Assistant Director of Nursing, Director of Education, Clinical Educator, Education Department, and Clinical facilitators. Collectively, we agreed on our communication pathways which were meetings, email, and informal briefings.

Having identified our stakeholders we began to gather what is known as the Voice of the Customer (VOC). The VOC involves speaking to those who work in, on, or with processes, or are impacted by them [47]. This was carried out as per our stakeholder agreed communication pathways. We initially conducted face-to-face informal interviews with staff and managers who were required to attend BLS training. These meetings identified potential NVA activity in the process coalescing around three main issues:

- Scheduling of staff for BLS training,
- The booking process for BLS training, and
- Time to complete training.

From our VOC sessions, we mapped the staff voice to a Critical to Quality (CTQ) [46] tool to help us identify the CTQ parameters as they relate to what is important to the customer at large. The CTQ indicated to us the importance of continually seeking the VOC (in this instance the ‘customers’ were the hospital staff), in reviewing the BLS training system itself and measuring the time taken to deliver the current end-to-end process for BLS training. Further VOCs were conducted through qualitative face-to-face interviews with a random sample of staff nurses (n = 10), ward managers (n = 10), and department heads (n = 10).

We focused our initial data capture and analysis on the attendance and certification figures for BLS. This indicated the increasing yearly demand for BLS due to organizational growth from 736 clinical staff in 2018 to 941 in 2020 a 22% increase in the number of staff trained per month and the year varied from between 16 and 69 staff per month. A review of the attendance between 2018 and 2020 at the first 20 classes per year demonstrated a large variance in the numbers of staff per class from 1 to 12. This led us to our measure phase.

### 3.2. Stage 2: Measure

We used the CTQ tool to translate the VOC obtained in the define phase into measurable metrics which were:

Metric 1: Time taken to complete scheduling,
Metric 2: Time taken to complete training, and
Metric 3: Staff with BLS expired due to lack of class availability.

We identified staff and BLS instructor surveys as a priority and we issued an online survey to both staff and managers. This showed 14% of staff (n = 100) responded including 3% of managers (n = 100) (Table 3). The VOC survey results indicated once again that the main issues identified surrounded scheduling, booking, and time (Table 3).

**Figure 1:**
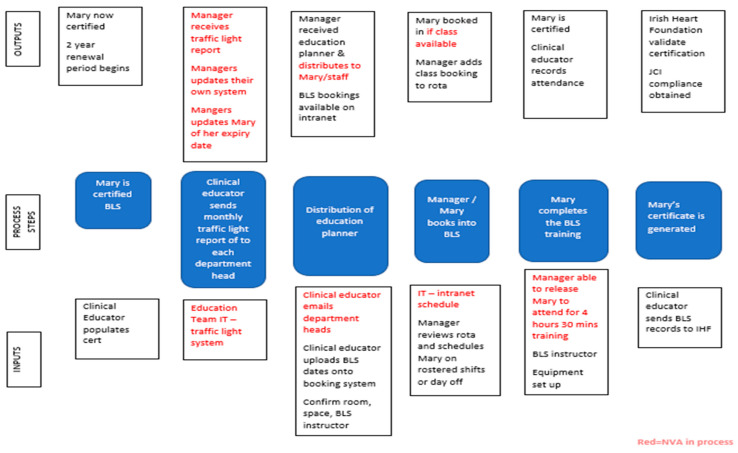
IPO Map.

Process mapping is a technique used to visualize, document, analyze and improve the flow of information or materials required to improve service for customers [46] and has been shown to be valuable in understanding the complexity of healthcare improvement interventions [48]. The team chose to utilize the IPO process mapping tool (Figure 1), that enabled us to identify the key steps for review in the process. Combining the results of our SIPOC and IPO enabled us to have an understanding of where the NVA occurred. For example, two scenarios that arose were staff who “did not attend” (DNA) and staff who “cannot attend” (CNA). These can directly impact staff completing their BLS after their actual license expiry date. From the completion of the IPO map (see Figure 1), the main issues of scheduling, booking, and time constraints were made visible.

Several observational Gemba’s (n = 3) were completed (Table 4). Gemba A measured the time it took for staff members to book a BLS class which took an average of 15 min to complete. We then proceeded to complete a scoping exercise with our key stakeholders to agree and clarify the intended scope of this study.

The interventions carried out in a study by Aij and Tunissen [42] mirror our use of LSS by utilizing a Gemba. Aij and Tunissen [42] highlight that the use of a Gemba is a component in Lean leadership and a critical problem-solving tool, and two Gemba’s were included as part of this project. Our team mirrored this approach and conducted a Gemba walk with time set aside solely for the Gemba walk. Aij and Tunissen [42] advise that during the Gemba walk, one must approach others with respect seeking input from others through Socratic questioning. This was carried out by three of our team members during our study.

#### 3.2.1. Scheduling

The number of scheduled classes per year ranged from 52 to 62 between 2018 and 2020. Of note, 5 classes were allocated to healthcare assistants (HCAs) only in 2020 (Table 5). When the team compared the number of classes available on the education planner with the number of scheduled classes per year, a disparity was noted. Unscheduled classes occurred where they were arranged to meet demand and ranged from between 32 and 88 staff members participating (Table 5). Upon review of the class attendance, it became apparent that unscheduled BLS classes were required to meet the demand within the organization and could have allowed for more attendees.

Gemba B (Table 4) was completed to ascertain the time spent by the clinical educator distributing the BLS expiry report monthly to department heads. This was measured at 320 min.

The clinical educator emails the education planner to department heads to communicate upcoming BLS classes. The release of the Education planner via email to managers ranged from 14.5 to 20 days which made it difficult for staff to schedule bookings as shifts were already rostered by the time the education planner was made available.

#### 3.2.2. Booking

Between 39% and 62% of staff attended training after the expiry date (Table 6). Further analysis of the number of BLS classes offered and staff attendance provided data on the underutilized capacity. This was attributed to as already outlined to staff who DNA, or to staff who CNA due to clinical needs requiring their clinical presence, which accounted for between 15% and 28% from 2018 to 2020. Considering reduced class capacity due to social distancing requirements arising from the COVID-19 pandemic, this placed additional stress on the capacity of the BLS instructors to deliver the demand for BLS certification.

Gemba C (Table 4) was completed whilst observing the delivery of two BLS classes delivered by two different BLS instructors. NVA was recorded between 66 and 82 min and this was due to the delivery method, which was in place at the time, a video-assisted simulation method of 4 to 5 h face-to-face training in class, and staff whom DNA and CNA (Table 6) leading to increased administrative workload, e.g., follow up emails, rescheduling staff, revising training rosters and dates.

#### 3.2.3. Cost

As nursing staff make up 50% of clinical staff in the organization average cost was based on this cohort’s payscale. The average cost for the organization per BLS class with 6 staff members in attendance was calculated as €523.26 on average. This equated to €87.21 per staff nurse in class on average. The BLS instructor time was a fixed cost to the organization.

### 3.3. Stage 3: Analyze

**Figure 2:**
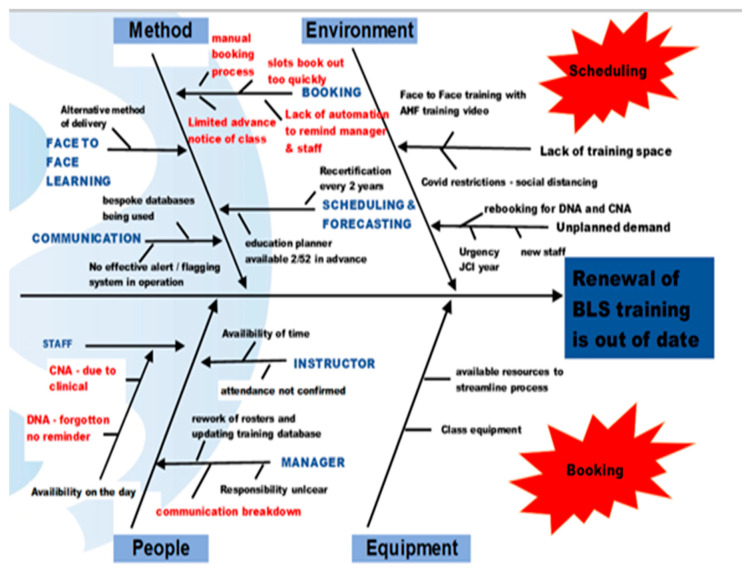
Fishbone Diagram.

To facilitate root cause analysis of our collated data, the team utilized a Fishbone diagram (Figure 2). Four categories of variation were defined: method, environment, equipment, and people. Two keywords became apparent in the use of this LSS tool scheduling and booking.

Scheduling: Using the Fishbone tool helped the team to identify that the release of the BLS class timetables was offered to staff after the staff rosters were confirmed by managers and department heads. This affected staffs’ ability to book BLS classes as they had to request time to complete training from already populated staffing rosters, and put pressure on the patient-to-staff ratio. Similarly, the delayed release date of BLS class timetables certainly led to an increase in rework for both ward managers and staff alike, as staffing schedules had to be amended and updated. This highlighted the impact on the staff’s ability to renew their BLS certification within the required renewal date.

Booking: Using the Fishbone tool identified that the current booking process was heavily dependent on manual input. To book a BLS class, the managers/staff needed to refer to multiple sources for information: BLS class timetable, staff training record, clinical educators traffic light system, staff roster, individual staff training profile, using multiple methods to access these including email, shared network drives, intranet, and paper-based files. This had become a multifactorial process that had been impacted by the rapid growth of the hospital. Many ward managers had added in their own bespoke processes to micromanage their staff’s mandatory renewal training in BLS, leading to the introduction of more variation into an already complex system.

Our analysis also revealed that there was a breakdown in the flow of communication throughout the steps of the process for releasing, scheduling, and booking BLS. This reflected our feedback from our VOC sessions that indicated the main contributing factor of the high number of CNAs was due to clinical demands and the DNAs was due to the lack of reminders issued to participating staff. To further analyze the root cause of the problem we posed two questions “Why do we have a high number of DNAs?” and “Why do we have a high number of CNAs?” therefore we used the 5 Why analysis tool [49,50], with our analyses arriving at the same root cause statement: —there are no controls in place for the current system and it is heavily based on manual input”. Concerning the CNAs, the 5 why analysis indicated that the booking and scheduling process was in its current state unfit for purpose. We also found that ward managers who released staff for BLS training had no capability for filtering the mandatory training data in real-time. Concerning the DNAs, the 5 Why’s questions indicated that there was no cancellation list available to ward managers, to enable them to avail of underutilized capacity. The ward managers could not adhere to the protected time policy which is allocated to and used by all staff to allow them time away from their clinical commitments to complete training and education.

At this stage, the team realized that we needed to delve deeper into the IPO. We drafted and populated an FMEA [50] with our findings, this is a systematized table of activities set out to recognize and evaluate the potential failure of a product or process. It identifies actions that could eliminate or reduce the occurrence of the potential failure and documents the process.

**Figure 3:**
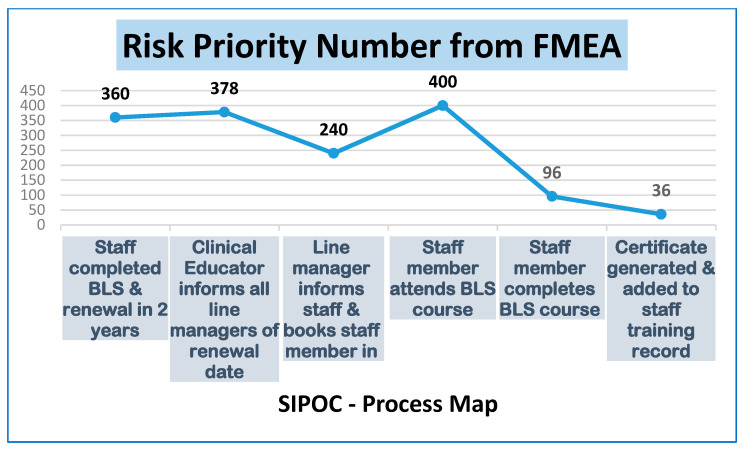
Risk Priority Number Report.

Our findings directed us to the two highest Risk Priority Numbers (RPN), this is the numeric assessment of risk assigned to each individual process plotted on the FMEA table, the two highest scores being 400 and 378 (Figure 3). These two highest scores refer to the following NVA points:

- RPN 400: The Staff member attends the class. The FMEA identified that there is no method of alerting the educator of any potential CNA or DNA, which could, in turn, affect the attendance rate, as there is no defined process in place to confirm or remind the staff member of their booked class. In tandem, from the manager’s perspective, they cannot edit or adjust their staff’s scheduled class, and they do not have the necessary access to pull or swap from a cancellation list. It was also identified that post-BLS training, there was no analysis or reporting of CNA or DNA.
- RPN 378: The clinical educator informs all line managers of their staffs’ BLS renewal date. Communication to the manager only delays communication to the staff member creating a failure mode. By this time the BLS Slots advertised have been booked out. This could result in the staff member working on the ward unaware that their BLS is out of date.

### 3.4. Stage 4: Improve

Based on our root cause analysis of the collated data, it was agreed with our stakeholders that the following interventions were to be deployed:

1. Analysis of the BLS report to forecast the number of staff requiring BLS training in the next year and forecasting of the number of classes (Table 7).
2. Redesigning of IT solutions for scheduling and booking of BLS were agreed as below:
  - Ward managers to have access to view their own individual mandatory training records and have access to view their ward staffs’ mandatory training reports. This removed the need for manual data entry to spreadsheets by managers and allowed ward managers to track their staff’s mandatory training.
  - To ensure capacity was fully utilized by relevant staff members and ward managers, they were reminded via an automated email sent by the Clinical Educator that their BLS would be expiring in the next three months. A week before the booked class staff member and ward managers also received an email reminder of the upcoming booking. A cancellation list was generated to fill empty spaces automatically.
  - Attendance at BLS was recorded automatically through staff signing in with a staff identification badge and a barcode reader. This reduced the manual process of BLS instructors updating staffs training records manually.
  - Automation of forecasting of upcoming BLS expiry dates to ensure BLS training classes meet demand.
  - Introduction of a Blended Learning Method (BLM) of BLS Heartcode training. BLS Heartcode is a self-directed, comprehensive eLearning program that uses adaptive learning technology to allow learners to acquire and demonstrate BLS Skills with a personalized learning path that adapts to the individual learner’s performance [50,51,52,53,54].

The sample size of the pilot represented 10% of the total number of staff requiring BLS training/recertification (n = 140). As of September 2021, 81 staff had utilized the new process to attend BLS. A meeting was organized with key stakeholders for the planning of the BLS Heartcode Pilot. This required the commitment of the BLS instructor to deliver the BLM and the team agreed to collate the evaluations. Buy-in was required by senior management to approve the process and a willingness from the education department to deliver the BLS Heartcode pilot. This method was chosen as it allows for a flexible learning environment for staff with the BLM.

BLS Heartcode allows users access to the e-learning component of BLS Heartcode known as part 1 and was obtained from the IHF. The hospital marketing team assisted in formatting and distributing a flyer that advised that the new BLS Heartcode process and availability of BLS classes, via internal email communication to all staff who were eligible to be part of the improvement project. Those eligible for BLS were staff for recertification of BLS. BLS Heartcode available training classes were released on the hospital IT booking system 3 months in advance and staff began to book into the sessions themselves. Communication was also released and targeted to all Heads of Departments around the BLS process change. The use of blended learning approaches is on the rise in healthcare for many reasons such as limitations in travel due to the current COVID-19 pandemic, social distancing requirements reducing numbers who can attend in-person training, general demands on staffing levels due to national shortages of healthcare workers especially within the nursing workforce. The literature surrounding the use of the BLS blended learning approach is not available; however, it has been widely used in healthcare education and has been shown that the blended approach to education is not only comparable but at times superior to the traditional face-to-face method due to enhanced students’ knowledge and problem-solving ability [53,54]. The use of blended learning allows the creation of flexible training when resources are limited [53].

## 4. Results

### 4.1. Pilot Results

The BLS Heartcode Pilot was introduced in June 2021 with increased scheduling in advance of available BLS classes from an average of 27 days to 60 days. Attendance between June, July, and August 2021 was 82 participants with 18 classes compared to the same period of 87 participants with 16 classes. The results have shown that before the process change the average time for staff (n = 12) was 4 h 5 min in Gemba C (Table 4). After the process change the total BLS training time was reduced to 2 h 9 min. This illustrates a total time saved for each staff member of 1 h 56 min, a 47% improvement in the time taken to complete the BLS process.

On review of the results of the BLS instructors’ time before the process change, the BLS instructors training and administration time was 5 h. After the process change, the time was reduced to 1 h 19 min. This was a time saving for the instructor of 3 h 41 min. This demonstrates a 74% improvement in the time taken to deliver the BLS. It was also noted that there has been a 50% increase in staff throughput for BLS training, with a projected forecast of 720 staff in 2022. This will allow a significant release of staff time for patient care. Additionally, feedback from the staff evaluations is that 64% of staff prefer the BLM.

The pilot results have led to the adoption of a new process for BLS booking, scheduling, and training. To date, 81 staff have completed the BLS under the new process. The IHF has a standard BLS classroom feedback form. This was provided to all staff who attended BLS training via the BLS Heartcode method (n = 81) with a 96% response rate. One hundred percent of attendees indicated that the online component prepared them to pass the BLS skills session. Furthermore, one hundred percent of staff indicated that they felt prepared to respond to an emergency because of the BLS skills they learned. 69% preferred the BLS Heartcode method. This highlighted the theory provided online was more efficient than, and was the preferred choice of staff, to the traditional face-to-face method. In the comments section of the evaluation forms, twenty-seven percent of staff identified time saved as a benefit. Post-intervention results are outlined in Table 8.

### 4.2. Stage 5: Control Plan

Implementation of BLS Heartcode showed a 3% decrease in expiry dates of staff attending. As of September 2021, the roll-out of the Heartcode BLS method is currently moving from the pilot to the deployment stage. On a long-term basis, it is envisaged that by the end of 2022 all staff will be re-certified in BLS before their expiry date continuously. To ensure the new process stayed within control we agreed with key stakeholders that the key metrics of DNA, CNA, and time spent scheduling and training would be monitored by the Education Team on an ongoing monthly basis.

## 5. Discussion

The study site has not only enabled but championed this process improvement project as its ethos is to continually improve processes and strive for excellence. The team was facilitated by LSS training opportunities to equip us with the knowledge and skills to complete our LSS journey with continual support provided by key stakeholders to enable the required data collection, analysis, and co-design of solutions. This pilot study using a LSS approach has demonstrated a reduction in the time to complete mandatory training without an additional resource requirement.

BLS Heartcode pilot has been shown to facilitate a cohesive, streamlined, and time-saving approach to BLS instruction and training. The Republic of Ireland’s BLS training is conducted through an online video-assisted learning tool which is governed by the IHF. The BLM of BLS Heartcode is available and accredited by the AHA for use internationally [48]. The requirement of BLS training as a mandatory training requirement for JCI has been historical across the private healthcare service, and therefore implementing changes were initially received cautiously by those working in the education department. However, this study demonstrates that with an effective study design and robust data collection, change for the progression of the organization, the staff, and most importantly the patient population is required.

The success of this study was in part due to the diverse team of professionals working on this study who were all undertaking the LSS training with our academic partner. The majority of team members had limited experience in mandatory education except for two members. The team had time provided to work in a structured and goal-orientated manner over a designated 9-month period using the LSS tools through a time in healthcare that was increasingly problematic because of the Global COVID-19 pandemic. However, it must be acknowledged the great support and engagement which was given by our senior management team, academic partners, and respective departments in supporting us to continue and complete our project. LSS training provides a set of team-building tools that help individuals to collaborate, engage and negotiate with a variety of disciplines across the organization in pursuit of change [54,55,56,57,58,59].

While change implementation can be challenging, the team found that aligning individual projects to the strategic goals of the organization and recognizing the voices of all the customers affected by the project is essential to sustaining LSS [52,53,54,55,56,57,58,59,60]. The team ensured to include key stakeholders to include: Chief Operations Officer, Director of Human Resources, Director of Nursing, Assistant Director of Nursing, Director of Education, members of the Education Department, and Clinical facilitators. This allowed for collaboration and buy-in for the BLS Heartcode Pilot project. Once approved at this forum, the BLS Heartcode Pilot was then approved by the financial team. Guidance and support from the LSS Black Belt supervisors greatly aided the success of change implementation in this study.

The learning from this LSS project for other hospitals is that implementing a blended approach to mandatory education can be applied to multiple types of training such as Manual Handling or Sepsis training. The next phase of our work is to enhance the scheduling and booking process for BLS. To date, we have met with the IT project coordinator and education departments at the study site to map out how the scheduling/booking process can be enhanced. There are further IT improvements ongoing to reduce the BLS instructors’ administration times.

The new streamlined process for BLS training has had implications for managers across the organization. At the senior management level, this emphasized budgeting for a yearly cost analysis of the online BLS Heartcode method. For our IT managers, it has meant frequent data analysis to provide a focus on BLS expiry dates on an ongoing basis. For our clinical governance team, there was a requirement for a policy change for BLS training that all staff must attend prior to their expiry date with clear inclusion and exclusion criteria. For ward and unit managers there was a change in process and a requirement for staff to be booked into BLS training in advance thus meaning that ward managers must complete rosters in advance. Finally, the training managers have had to provide extra support in each area to enable efficient scheduling of training. However, LSS as a process improvement methodology has a focus on developing the capabilities of teams (doctors, nurses, and administrative and support staff) to manage and continuously improve their work [61]. As a learning organization, the study site also recognizes that when an organization begins to adopt LSS, individual and team-based learning is the focus, not just in the classroom, but in the practice area [3].

A strength of this study was that it consulted directly with staff, capturing their perspective, sources of information, and through the use of customer voice [48], their specific areas of need. However, we recognize that this study was not without limitations. Time limitations were a challenge for all team members and not readily anticipated from the outset with the unprecedented Global Sars-Cov pandemic, team members working remotely and redeployed. It was increasingly difficult to take time from clinical hours to conduct team meetings. There was a lack of published research specific to the use of both LSS methodologies concerning mandatory training to draw on for this study. However, the literature reviewed and critiqued by our team has highlighted to us how the LSS methodology and concepts can significantly contribute to identifying NVA and enhance process improvement [6,14,21,22,24,27]. We acknowledge that this was a pilot study within a single site location. We recognize that as a pilot study, we could only examine feasibility of the training type (BLS) included in this study and within the study site. The results do not necessarily generalize beyond the criteria of the pilot. However, as pilot studies are conducted to evaluate the feasibility of some crucial component(s) of a full-scale study, we believe it has implications for other hospital sites and their academic partners who may wish to explore this question as a full study. The use of LSS facilitated the improvement of BLS mandatory education. Learning for the team was that drawing on the experience, knowledge, and expertise of staff members educated and trained in LSS can have many benefits to the project. Future studies could be carried out to evaluate the implemented improvements and further studies on education department processes using the LSS approach.

## 6. Conclusions

We identified an issue with staff education and licensing in BLS. By conducting a pre- and post-team-based intervention using a LSS approach, we redesigned the existing process for BLS training to enable staff to attend before their license expiry date and implemented a new method of BLS education delivery, which in its pilot phase has enhanced staff satisfaction with 64% of staff preferring the blended learning approach. This allowed a 50% increase in the amount of BLS training being offered. The new method also allowed for healthcare staff time released to contribute to increased nursing time with their patients and reduced risk to the organization by ensuring relevant healthcare staff are certified to carry out BLS in an emergency situation before their expiry. Another implication of this change has been a saving of time for the actual training itself, releasing further staff time to care. Importantly, it also ensures that the hospital has a staff base who are licensed to carry out BLS in the event of emergencies involving their patients.

To summarize the results of this study to date:

- Increased staff satisfaction with BLS training.
- Increase in the number of BLS classes that can be scheduled.
- Time saved for staff and time saved for the BLS instructors and Education department.

Joosten and colleagues [62] note the importance of linking process improvement to respect for the individual. The involvement of our stakeholders at all stages of the process redesign, from conception to pilot results, and our use of co-designed solutions aligns with a more person-centered culture that delivers person-centered coordinated care, a priority for healthcare stakeholders internationally. We found that the approach to the use of LSS within the study site was synergistic with the concepts of respect for persons and staff empowerment, themselves enablers of person-centered cultures [61]. The development of staff through support and respect is important for their engagement with LSS [2,3], and staff involved in this project advised they felt respected. We also experienced that the approach of giving to employees opportunities for development through the LSS education and training program within the hospital, rather than getting something from them, such as more productivity [63] is also synergistic with the person-centered value of respect for persons, which is enabled by empowering cultures [61].

The change process can be challenging for all people involved, the change agent and the participants involved in the change. We note that a more scientifically rigorous approach to the development, evaluation and dissemination of quality improvement methodologies such as LSS is ongoing [1,2,3,64]. From our review of the literature, we found multiple studies of LSS use in supporting clinical and support processes within the health system internationally [20,27,28,29,39,40,41,47]. However, as we reported when discussing limitations, we found a dearth of material relating to LSS use in healthcare mandatory training redesign. We therefore suggest that this study makes an important contribution to the body of evidence as to the wider use of LSS in healthcare outside of clinical processes alone. Importantly, this study also contributes to the growing body of work on the synergistic use of LSS and person-centered approaches to improvement [2,3,29,42,45,64,65]. Our person-centered focus on valuing our study participants and their values from the start, were synergistic with the philosophical intentions of person-centeredness [2,3]. This paper demonstrates that applying LSS and person-centered methodologies to processes for training that may at first seem far removed from ‘persons’ but are actually person dependent can yield results for all stakeholders at the levels of patient, staff, and organization. We therefore contend there is learning for healthcare staff and educators in many contexts within this paper.

## Figures and Tables

**Table 1:** LSS Tools.

Title of Improvement Tool	Definition	Output
Project charter	A project charter is used to define the problem statement and attain baseline data for the project.	It was useful in clearly identifying the goals of the project and what was in scope
SIPOC	Suppliers, inputs, processes, outputs, customers (SIPOC) is a tool that summarizes the inputs and outputs of one or more processes in table form [15].	This provided us with a simple and high-level view of the process and its elements
CTQ	Critical To Quality Tree [15].	Identifies the needs and drivers of the stakeholders and/or process that is critical to achieving quality
RACI	Responsible, accountable, consulted, and informed—identifies which stakeholders were responsible, accountable throughout the DMAIC and which stakeholders needed to be kept informed or consulted	This table defined and mapped out the roles and who was responsible for each action item
IPO	Input-Process-Output—associated with a diagram that visually represents the process with inputs shown on the left and outputs shown on the right. Assists in understanding proactive and reactive improvement. Strive for addressing the inputs to a process.	
Gemba	Comes from the Japanese phrase ‘genchi genbutsu’ meaning go and see and specifically means ‘the actual place’ [10,15,16,17].	A Gemba is where the actual place where the process takes place in the workplace is observed
Ishikawa diagram is also known as a Fishbone diagram	A visualization tool for categorizing the potential causes of a problem. This tool is used to identify a problem’s root cause, a Fishbone diagram combines the practice of brainstorming with a type of mind map template to determine cause and effect [16].	To determine the cause and effect of a problem
5 Why	5 Why’s root cause analysis asks the question ‘why’ as many times as necessary to identify why a problem has occurred or what the root cause is.	To identify the root cause of a problem
FMEA	Failure mode and effect analysis is a systematic, proactive method for evaluating a process to identify where and how it might fail and to assess the relative impact of different failures.	To identify the parts of the process that are most in need of change
Control plan	A live document that outlines the methods taken for quality control of critical inputs to deliver outputs that meet customer requirements.	Provided a written description of the measurements, inspections, and checks put in place to control each stage of the process

**Table 2:** Monthly classes 2018–2020.

	2018	2019	2020
Monthly classes average	4.3	5.2	4.9

**Table 3:** VOC Survey Results.

(N = 100)	Scheduling
74%	Of managers use email to flag expiry dates, many managers have created their own flagging system, staff boards, and spreadsheets.
30%	Of managers found that planning, maintaining, and updating their staff training records the most challenging.
	**Time**
45%	Of managers found releasing staff the most challenging stage of the renewal process.
43%	Of staff have not been able to attend their scheduled BLS training
39%	Unable to attend due to having staffing and cover issues.
	**Booking**
50%	Of staff have elapsed their renewal date due to lack of class availability.

**Table 4:** Gemba.

**Gemba**	**Title**
Gemba A	Gemba of a staff member scheduling BLS
Gemba B	Gemba of Education Report
Gemba C	Gemba of BLS Delivery

**Table 5:** BLS classes.

	2018	2019	2020
Number of scheduled classes	52	62	59
Staff attendance at scheduled classes	349	41	327
Staff attendance unscheduled	75	88	32

**Table 6:** BLS training breakdown.

	2018	2019	2020
BLS completed after the expiry date	N = 263	N = 504	N = 359
Percentage completed BLS after the expiry date	62%	39%	54%
Average days completed after the expiry date	136	55	74
CNA/DNA	26%	28%	15%

**Table 7:** Forecasted of required BLS training.

	2021	2022	2023
Number of staffs BLS Expiring	305	424	504
Number of classes required	50	70	84

**Table 8:** Post Intervention Results.

(N = 113)	Scheduling
71%	81 members of staff scheduled for Heart Code BLS attended
	**Time**
19%	21 respondents to the Heartcode evaluation form stated that this method saved them time
21%	24 staff did not attend Heartcode BLS (Pilot)
8%	8 staff members could not attend Heartcode BLS (Pilot)
	**Booking**
47%	38 staff members who attended BLS Heartcode certification had expired before attending

## Data Availability

Access to the BLS Heartcode keys was obtained from the Irish Heart Foundation. Information about this product can be accessed at resus@irishheart.ie.
